# Kosciusko County Medical Society

**Published:** 1849-01

**Authors:** 


### ARTICLE II.

### KOSCIUSKO COUNTY MEDICAL SOCIETY.

We have received a notice of the organization of the above named association from Dr. E. R. Parks, Corresponding Secretary to it; together with two papers for publication, which were read before it: which show that they have commenced operations in the right way, and are not disposed to put the candle they have lighted under a bushel. The officers for the current year are, Dr. R. Willard, President; Dr. Geo. W. Stacy, Sec'y; Dr. Wm. Parks, Corresponding Sec'y; Drs. R. Willard, G. W. Stacy, and W. S. McBride, Censors.

The notice holds the following language, "A very deep interest is felt by the members of this society for the advancement of our science, and the general interests of the profession."

We heartily wish them success in their laudable enterprise.

